# Unique quadruple immunofluorescence assay demonstrates mitochondrial respiratory chain dysfunction in osteoblasts of aged and PolgA^−/−^ mice

**DOI:** 10.1038/srep31907

**Published:** 2016-08-24

**Authors:** Philip F. Dobson, Mariana C. Rocha, John P. Grady, Alexia Chrysostomou, Daniel Hipps, Sharon Watson, Laura C. Greaves, David J. Deehan, Doug M. Turnbull

**Affiliations:** 1 Wellcome Trust Centre for Mitochondrial Research, Institute for Neuroscience, Medical School, Newcastle University, United Kingdom; 2 Musculoskeletal Research Group, Medical School, Newcastle University, United Kingdom; 3 MRC/BBSRC Centre for Ageing and Vitality, Newcastle University, United Kingdom; 4 Institute of Cellular Medicine, Newcastle University, United Kingdom

## Abstract

Fragility fractures caused by osteoporosis affect millions of people worldwide every year with significant levels of associated morbidity, mortality and costs to the healthcare economy. The pathogenesis of declining bone mineral density is poorly understood but it is inherently related to increasing age. Growing evidence in recent years, especially that provided by mouse models, suggest that accumulating somatic mitochondrial DNA mutations may cause the phenotypic changes associated with the ageing process including osteoporosis. Methods to study mitochondrial abnormalities in individual osteoblasts, osteoclasts and osteocytes are limited and impair our ability to assess the changes seen with age and in animal models of ageing. To enable the assessment of mitochondrial protein levels, we have developed a quadruple immunofluorescence method to accurately quantify the presence of mitochondrial respiratory chain components within individual bone cells. We have applied this technique to a well-established mouse model of ageing and osteoporosis and show respiratory chain deficiency.

The pathogenesis of declining bone mineral density, frequently culminating in a diagnosis of osteoporosis, is poorly understood. Following attainment of peak bone mass at approximately 30 years of age, a decline of bone mineral density ensues and continues throughout life unabated, universally affecting men and women^1^,^2^. Bone strength gradually diminishes as mineralisation levels fall and bone porosity increases, the consequence of which is an increasing risk of fragility fractures with advancing age^3^,^4^. Fragility fractures are associated with significant morbidity, mortality and huge costs to healthcare economies worldwide^5^,^6^,^7^.

Current theories on the underlying causes of declining bone mineral density include falling production of sex hormones^8^,^9^, increasing production of endogenous glucocorticoids^10^,^11^, reducing activity levels and falling levels of insulin-like growth factor I^12^. Although key hormonal changes such as postmenopausal oestrogen loss clearly influence the dynamics of bone metabolism, changes that occur with age at a cellular level are probably underestimated.

A growing body of evidence in recent years has suggested a causative link between mitochondrial dysfunction and the ageing process^13^,^14^,^15^. In humans, somatic mitochondrial DNA (mtDNA) mutations accumulate with age and cause respiratory chain dysfunction in individual cells. These changes have been detected in both post-mitotic tissues such as brain, muscle and heart tissue, and in mitotic tissues such as the colon^16^,^17^,^18^,^19^,^20^,^21^. No studies have documented whether respiratory chain deficiency associated with ageing occurs in bone cells, likely due to the absence of a reliable method of assessment.

Mitochondrial mutator mouse models provide the strongest evidence of a causative link between mitochondrial dysfunction and an ageing phenotype, including osteoporosis. The polymerase gamma (PolgA^−/−^) mutator mouse has a mutation in the proof reading domain of the mitochondrial DNA polymerase which leads to the accumulation of mitochondrial DNA mutations at a much faster rate than in wild type counterparts. The prematurely aged phenotype includes a significantly reduced bone mineral density compared to age matched wild type mice^22^. There is further evidence for potential mitochondrial involvement in osteoporosis, for example in mice with mitochondrial transcription factor A (TFAM) deficient osteoclasts, increased resorption is observed, tipping the net balance in favour of bone resorption rather than formation^23^. In addition, mouse models with either a complete or osteocyte specific knockout of superoxide dismutase (Sod2), an enzyme involved in limiting oxidative stress in mitochondria, develop osteoporosis prematurely^24^,^25^.

Assessment of mitochondrial respiratory chain dysfunction in individual cells in other tissues such as skeletal muscle, myocardium, brain and intestine is well documented. The classical method for assessing the presence of complex IV defects in individual cells, established in 1989, is sequential cytochrome *c* oxidase (COX) and succinate dehydrogenase (SDH) histochemistry^26^. COX/SDH histochemistry is applied to unfixed, frozen tissue sections and results in variable staining intensity depending on the degree of deficiency of the targeted respiratory chain components. In the presence of COX, tissue stains brown and when completely deficient, only the blue SDH staining is visible. Quantification using this method is challenging however, with notable inter- and intra-observer variability in classifying the fibres by colour intensity^27^.

In our endeavours to characterise respiratory chain dysfunction at a cellular level in bone tissue, we found the application of COX/SDH histochemistry to frozen mouse bone to be a challenging method due to loss of tissue morphology and difficulty in identifying individual cell types. We have therefore adapted a method developed for the assessment of mitochondrial respiratory chain components in muscle tissue sections^28^ for use in bone tissue sections. This new method utilises a quadruple immunofluorescence technique that enables the simultaneous targeting of a cell marker and multiple mitochondrial epitopes, such that the respiratory chain protein expression of complexes I and IV can be quantified relative to mitochondrial mass in specific bone cell subtypes.

## Method

### Tissue samples

Mitochondrial mutator mice (PolgA^−/−^) were generated that had a knock-in missense mutation (D257A) in the second endonuclease proofreading domain of the PolgA catalytic subunit of the mtDNA polymerase, using a C57BL/6 background mouse^15^. Bone tissue from 11-month homozygous PolgA^−/−^ mice (n = 9) and wild type littermates aged 4 (n = 5) and 11 months (n = 8) was compared. All animal experimental procedures were conducted in accordance with the UK Home Office guidelines and under its approval (licence 60/4540).

### Tissue preparation

Whole femurs were dissected from mice and immediately fixed in 10% normal buffered formalin (Sigma Aldrich) for 72 hours at room temperature. The bones were then decalcified for 21 days at 4 °C in 14% tetra-EDTA (Affymetrix), the pH of which was adjusted to 7.4 using glacial acetic acid. The decalcification solution was changed three times a week during this period.

The tissue was then embedded in paraffin using a standardised automated process at our institution following which, 4 μm-thick longitudinal sections were cut and mounted on SuperFrost glass slides; these were allowed to air dry for a week.

Frozen, calcified mouse femurs from the same animals were used for COX/SDH histochemistry. Whole femurs were embedded in OCT and 6 μm-thick sections were cut using a Leica Cryojane tape transfer system. The tissue sections were left to air-dry for one hour prior to performing COX/SDH histochemistry.

### Dual COX/SDH histochemistry

The COX medium was prepared by combining 200 μl of cytochrome *c* stock solution (500 μM cytochrome *c* in 0.2 M phosphate buffer, pH 7.0) with 800 μl of 3,3′diaminobenzidine tetrahydrochloride (DAB) (5 mM DAB in 0.2 M phosphate buffer, pH 7.0) and few crystals of 20 μg ml^−1^ catalase. The SDH medium was prepared by combining 100 μl of sodium succinate stock solution (1.3 M sodium succinate in 0.2 M phosphate buffer pH 7.0), with 100 μl phenazine methosulphate (PMS) stock solution (2 mM PMS in 0.2 M phosphate buffer pH 7.0), 10 μl sodium azide stock solution (100 mM sodium azide in 0.2 M phosphate buffer pH 7.0) and 800 μl of NitroBlue tetrazolium (NBT) stock solution (1.5 mM NBT in 0.2 M phosphate buffer pH 7.0).

The air-dried sections were incubated in COX medium for 40 minutes at 37 °C, washed twice in PBS, then incubated in SDH medium for 30 minutes at 37 °C. Tissues were dehydrated through graded ethanol series and mounted using DPX. Brightfield images of sections were obtained using a Zeiss Axioimager M1.

### Quadruple immunofluorescence in bone tissue

Formalin-fixed, paraffin-embedded sections (FFPE) were dewaxed at 60 °C for 30 minutes then deparaffinised using 2 changes of xylene (Histoclear) and rehydrated through graded ethanol series (100% to 70%). The sections were then immersed in 70% ethanol with 0.25% ammonium hydroxide for 1 hour to reduce autofluorescence, before rehydration was continued in 50% ethanol for a further 10 minutes.

Antigen retrieval was performed in a 1 mM tetra-EDTA (Affymetrix) buffer, pH 8.0 at 80 °C for 30 minutes before immediate transfer to phosphate buffered saline (PBS). Sections were then incubated with 10% normal goat serum (in PBS) for 1 hour at room temperature (RT). Mitochondrial endogenous biotin activity was blocked using an avidin and biotin blocking kit (Vector laboratories) as per the manufacturer’s instructions prior to application of Vector’s mouse-on-mouse blocking kit.

Respiratory chain complex subunits were detected using mouse monoclonal primary antibodies against NADH dehydrogenase [ubiquinone] I beta subcomplex subunit 8 (NDUFB8) of and cytochrome *c* oxidase (COX) subunit 1 (COXI). These were applied in combination with a monoclonal antibody against the outer mitochondrial membrane protein porin (VDAC1) (all at concentrations of 1 in 100). Porin is a nuclear-encoded, voltage gated ion channel present in abundance in the mitochondrial membrane, and its presence serves as a marker for mitochondrial mass^29^. NDUFB8 is a nuclear-DNA encoded subunit of Complex I^30^ and COX-I is a mtDNA-encoded subunit of Complex IV^31^. An antibody to osteocalcin to detect osteoblasts is combined with the mitochondrial antibodies at a concentration of 1 in 50. Alternatively, an antibody to cathepsin k or sclerostin to detect osteoclasts or osteocytes respectively can be combined. Sections were incubated overnight in primary antibodies at 4 °C.

Following PBS washing, sections were incubated for 2 hours at 4 °C with the following secondary antibodies at a concentration of 1 in 200: 405 Dylight (Jackson) to porin, Alexa 488 (Life Technologies) to COX-I, biotinylated anti-IgG1 (Jackson) to NDUFB8 and goat anti-rabbit Alexa 546 (Life Technologies) to target the cell marker. Sections were then incubated for a further 2 hours at 4 °C in streptavidin-conjugated Alexa 647 (Life Technologies) at a concentration of 1 in 100, before wash steps and mounting using Prolong Gold (Life Technologies).

No primary control sections were prepared concurrently using duplicates of each section, omitting application of primary antibodies (apart from the cell marker), in order to enable subsequent detection and subtraction of background signal.

### Imaging

Imaging was performed using a Nikon A1 confocal microscope at 60× optical magnification with a 1.55× digital magnification applied, using 405, 488, 546 and 647 nm wavelengths. Sections from wild type mice aged 4 months with primary and secondary antibodies applied, were used to calibrate laser power settings in each of the 4 channels such that the pixel intensity was set with care taken to avoid any areas of under or over pixel saturation within a pixel intensity range of 0 to 4095.

### Image analysis

Imaris image analysis software (Bitplane, v.8.4) was used to automatically detect osteoblasts (546 nm) and areas within these that were positive for the mitochondrial mass marker porin (405 nm). For each of these areas of intracellular mitochondrial staining, the software provided average signal intensity values for porin (405 nm), COX-I/MTCO1 (488 nm) and Complex I/NDUFB8 (647 nm). An average signal intensity value for each of these components across the whole osteoblast cell surface was provided by the software.

In order to calculate non-specific background fluorescence, corresponding no primary control sections were used to obtain average signal intensity levels for 405, 488, and 647 nm wavelengths, using the whole cell surface.

### Data analysis

The average COX-I, NDUFB8 and porin signal intensities detected within mitochondrial syncytium of each individual osteoblast was recorded. For each channel, the average non-specific background fluorescence measured in the corresponding no primary control section was subtracted. These background corrected values were log transformed, and normality was verified in the 4-month control mice using the Anderson-Darling test.

Data from 4 month old wild type animals were used as the reference control. The following methodology has been previously reported^28^. Background corrected log transformed values are used throughout. In brief, 100 osteoblasts were randomly sampled from each control animal, pooled, and a single mean and standard deviation of porin intensity for the controls was established. The mean and standard deviation were used to derive a Z-score for the porin level in each osteoblast (all animals; 4 month wild type, 11 month wild type, and 11 month PolgA^−/−^).

Linear regressions of COX-I vs porin and NDUFB8 vs porin were also performed on the same pooled control data to yield regression estimates. The linear regressions were used to generate Z-scores for the COX-I and NDUFB8 intensity in each osteoblast based on their expected intensity according to their porin intensity (again, all animals).

Osteoblasts were classed as having normal levels of NDUFB8 and COX-I if Z-scores were no more than −3SD from the mean, intermediate (+) if Z-scores were between −3SD and −4.5SD, intermediate (−) if Z-scores were between −4.5SD and −6SD, and deficient if Z-scores were more than −6 SD below the mean. The level of intracellular porin detected was also classified according to Z-scores as “very low” if below −3SD,“low” if between −3SD and −2SD, “normal” if between −2SD and +2SD, “high” if between +2SD and +3SD, and “very high” if above +3SD.

All statistical analysis was performed using R^32^. Graphs depicting COX-I and NDUFB8 levels were generated using a web application provided by Wellcome Trust Centre for Mitochondrial Research (http://research.ncl.ac.uk/mitoresearch/).

## Results

### COX/SDH Histochemistry

**Figure 1:**
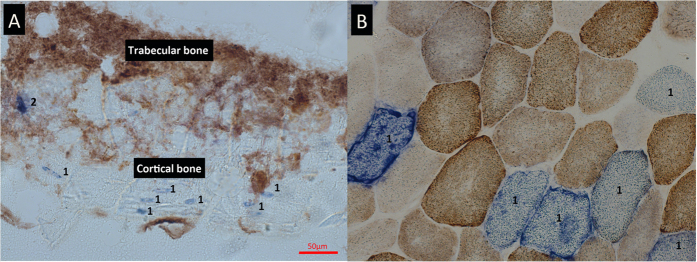
COX/SDH histochemistry of mouse femur. (**A**) Application of sequential COX/SDH histochemistry to mouse femur at 20x magnification. The process of cutting frozen sections from bone causes tissue destruction and loss of morphology. The COX deficient cells seen within cortical bone (1) are almost certainly osteocytes based on their shape and location. However, identifying other cell types, especially those residing in trabecular bone (2) is almost impossible using this method. (**B**) In contrast, frozen sections of muscle retain their morphology and clear delineation of individual fibres can be seen. In this example, several fibres (1) demonstrate COX deficiency with the remaining fibres showing variable intensities of COX staining.

COX/SDH histochemistry on bone tissue is shown in Fig. 1. Due to the tendency of calcified bone to crumble when cut, obtaining high quality frozen tissue sections is difficult, with substantial tissue destruction and loss of morphology frequently occurring, despite the use of a tape transfer system. Following COX/SDH histochemistry, accurate delineation of individual cells and identification of cell type is difficult, with cells within trabecular bone frequently overlying each other. This complicated the assessment of COX deficiency and made it challenging to obtain meaningful assessment of respiratory chain deficiency.

### Quadruple immunofluorescence

#### Optimisation of fixation and decalcification for immunofluorescence

Different fixation times using 10% NBF in combination with different decalcification methods were trialled (Supplementary Table S1). Fixation times of 24, 48 and 72 hours were combined with decalcification in 14% tetra-EDTA for either 18 days or 25 days at either room temperature or 4 °C. The same fixation times were also combined with decalcification using 10% formic acid for 15 hours at room temperature. Analysis of signal-to-noise ratios after implementing the immunofluorescence protocol described was performed. In tissue fixed for 24 or 48 hours, a drop in signal to noise ratio when decalcification temperature was increased from 4 °C to RT in EDTA was observed. There was no notable drop in signal or increase in background fluorescence when tissue was fixed for 72 hours despite prolonged decalcification time or increased temperature. There was no significant variability in signal to noise ratios with different fixation times when tissue was decalcified in formic acid but increased and detrimental levels of autofluorescence at microscopy were observed, particularly at emission wavelengths of 405 and 488 when 10% formic acid was used.

#### Imaging of bone tissue

**Figure 2:**
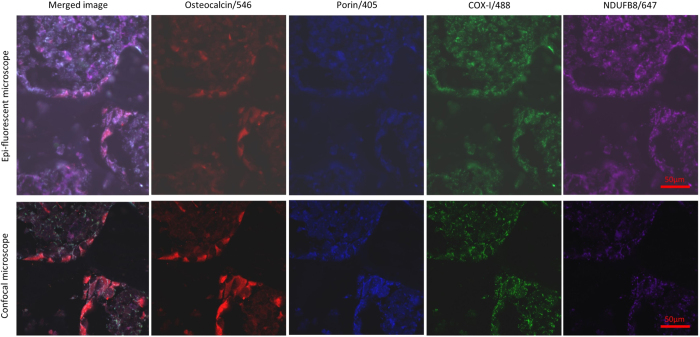
Image capture using epifluorescent light source vs confocal laser, 40x magnification. Contrasting results of imaging can be seen when an epi-fluorescenct light source is used (top panel) compared to a laser point scanning confocal microscope (bottom panel), images taken from the same area of the same tissue section. Confocal microscopy is well known to confer several advantages over conventional microscopy. We found it to provide better clarity of target florophores with clear punctate signal emanating from target mitochondrial epitopes and lower levels of autofluorescence (as also demonstrated in Fig. 3).

Immersion of tissue sections in a 0.25% ammonium solution for one hour reduced the degree of autofluorescence to satisfactory levels and was particularly effective in the 405 and 488 nm emission wavelengths as per the findings of previous work^33^. The use of a microscope with an epi-fluorescent light source gave suboptimal images with very poor signal to noise ratios and high levels of autofluorescence. The use of a laser point scanning confocal microscope dramatically improved resolution and reduced levels of autofluorescence (Fig. 2). To minimize autofluorescence laser power had to be minimized, thus careful pairing of secondary and primary antibodies was required. As the highest levels of autofluorescence are present in the 405 wavelength, we found that anti-VDAC1 was the only antibody able to provide an adequate signal to noise ratio, presumably because of the high amount of substrate its target epitope provided. We were not able to visualise cell markers, COX-I or NDUFB8 using a 405 secondary antibody. In order to generate adequate signal in the 647 wavelength, it was necessary to biotinylate the anti-NDUFB8 antibody. Again, porin was the only antibody possible to visualise in this channel without augmenting the signal with a biotinylation step. The cell markers were clearly visualised in the 546 wavelength with no problematic autofluorescence observed. Due to the high levels of endogenous biotin contained within mitochondria, and because a streptavidin conjugated secondary was being used to detect NDUFB8, the use of an avidin biotin blocking kit was required.

#### Application of optimised assay to bone tissue

**Figure 3:**
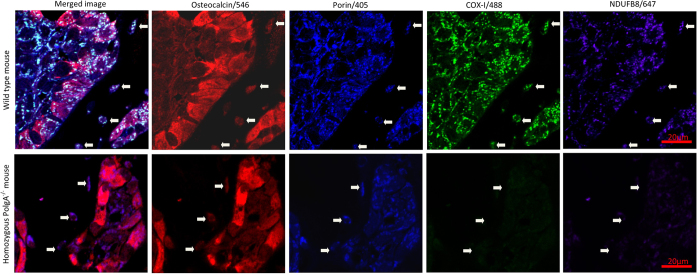
Application of quadruple immunofluorescence assay to mouse femur, 90x magnification. Images of bone lining osteoblasts targeted with an antibody to osteocalcin, imaged at 90x magnification using confocal laser microscopy. In comparison with aged matched control mice (upper panel), NDUFB8 (Complex I) and COX-I (Complex IV) mitochondrial respiratory chain deficiencies are seen at 11 months in PolgA^−/−^ mice section (lower panels) relative to mitochondrial mass (porin). The white arrows demarcate osteocytes seen within the adjacent cortical bone; the same deficiency is clear to see in these cells also.

**Figure 4:**
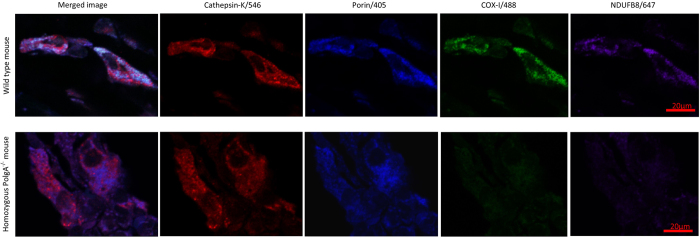
Application of quadruple immunofluorescence assay to mouse osteoclasts, 90x magnification. Application of optimised assay to mouse osteoclasts using antibody to cathepsin K, imaged at 90x magnification using confocal laser microscopy. Age matched wild type control mice demonstrate much higher signal intensities for NDUFB8 and COX-I in relation to porin levels (top panel) when compared to 11 month PolgA^−/−^ mice in this example (lower panel).

**Figure 5:**
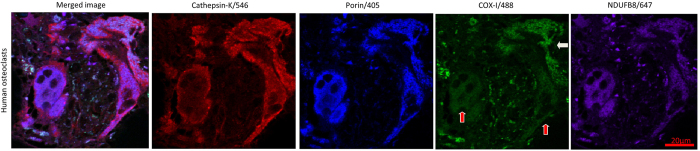
Application of quadruple immunofluorescence assay to human bone, 90x magnification. The method also detects mitochondria in human osteoclasts with an antibody to cathepsin K, imaged at 90x magnification using confocal laser microscopy. COX-I deficiency of the osteoclasts labelled with red arrows can be seen relative to mitochondrial mass/porin signal. The osteoclast labelled with a white arrow displays normal levels of COX-I.

Application of the quadruple immunofluorescence protocol to FFPE mouse bone tissue sections allowed preservation of excellent morphology and accurate delineation of osteoblasts, osteoclasts and osteocytes as well as clear demarcation of mitochondrial syncytium within individual cells, and identification of COX-I and NDUFB8 activity (Figs 3 and 4). We have also successfully applied the same assay to human bone to visualise osteoclasts using small sections of femoral head which had been processed in the exact same manner as the mouse femurs (Fig. 5).

#### Application of optimised assay to mouse osteoblasts

**Figure 6:**
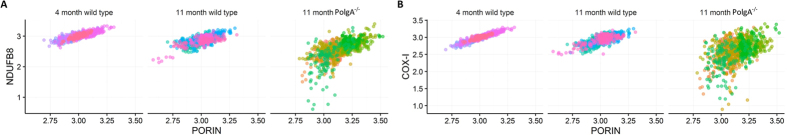
NDUFB8:Porin and COX-I:Porin ratios in mouse osteoblasts. Following log transformation of background corrected signal intensities for porin, COX-I and NDUFB8, linear association of NDUFB8 to porin (**A**) and COX-I to porin (**B**) are evident. The strongest association exists in the youngest wild type mice at 4 months when respiratory chain deficiencies are not expected to be present at significant levels. A moderate loss of this association is seen in older wild type animals, whilst the relationship is notably weak in the PolgA^−/−^ osteoblasts at 11 months.

Wild type mice aged 4 months (n = 5) and 11 months (n = 8) were compared to 11 month PolgA^−/−^ mice (n = 9). The relationship between mitochondrial mass (porin), COX-I and NDUFB8 protein abundance in the 4 month-old mice was linear. The relationship was also linear in 11 month wild type mice though with visibly higher variation, and notably more variable in 11 month PolgA^−/−^ animals with many cells displaying low COX-I and NDUFB8 protein abundance (Fig. 6).

**Figure 7:**
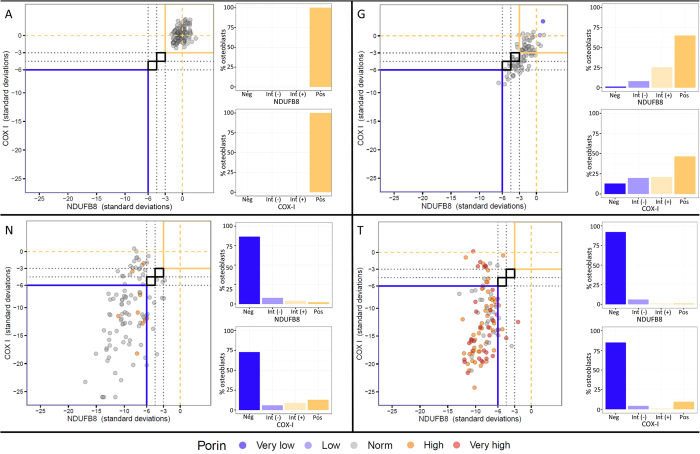
Representative graphs of Z-scored NDUFB8:Porin and COX-I:Porin for wild type and PolgA^−/−^ mice. The mean and standard deviations of NDUFB8:porin and COX-I:porin relationships in the 4 month wild type controls are established to derive Z–scores for porin, NDUFB8 and COX-I. Z-scores for NDUFB8:porin and COX-I:porin are plotted against each other for individual mice (main graph on left of each panel). Each dot represents a single osteoblast, colour coded by the porin level (dark purple, very low; light purple, low; grey, normal; orange, high; red, very high). Typical appearances of data for 4 month old wild type mice (**A**), 11 month wild type mice (**G**), and 11 month old PolgA^−/−^ mice (**N,T**) are shown (lettering corresponds with that in Table 1). At 4 months in wild type animals, the vast majority of data points are no more than 3SD from the mean of this young control group. Reduced NDUFB8 and COX-I protein expression is seen in 11 month wild type animals and to a greater degree in 11 month PolgA^−/−^ mice. The corresponding percentage of osteoblasts which are positive, intermediate positive, intermediate negative or negative for each mouse are also shown on the right side of each panel, for NDUFB8 (top) and COX-I (bottom).

Compared to the 4-month old wild type mice, 11-month wild type mice demonstrated COX-I levels 1.8SD lower on average (p = 0.136) and NDUFB8 levels 1.9SD lower on average (p = 0.014). The 11 month PolgA^−/−^ mice displayed mean COX-I levels 13.4SD lower (p < 0.0001) and NDUFB8 levels 8.2 SD lower (p < 0.0001) when compared to 4 month wild-type counterparts. When age-matched wild type and PolgA^−/−^ mice were compared (both 11 months old), COX-I and NDUFB8 levels in the latter were found to be 5.6SD and 3.9SD lower respectively (p < 0.0001). Numerical data for individual mice is shown in Table 1. Corresponding individual graphs for all mice, depicting NDUFB8:porin and COX-I:porin ratios following Z-score analysis, which clearly show deficiency type and severity are provided in Supplementary Data S2. Figure 7 shows representative examples of these graphs for 4 month old wild type, 11 month old wild type and 11 month old PolgA^−/−^ mice.

## Discussion

As humans age, somatic mitochondrial DNA mutations accumulate^13^,^17^ and there is evidence that this may be causative in the ageing process, as suggested by mitochondrial mutator mouse models^22^. However, changes at a cellular level in bone have never been demonstrated previously using a quantitative method. COX/SDH histochemistry has been the gold standard of quantifying respiratory chain deficiency in individual cells for many years^26^, and has been successfully applied to various tissue types. Our attempts to apply this method to bone tissue have shown it to be an ineffective method for this tissue with several disadvantages. The method is only successfully applied to unfixed tissue sections; however, whilst tissues such as muscle retain excellent morphology in the form of frozen sections, it is extremely difficult to obtain sections of undecalcified bone from frozen tissue without substantial tissue destruction^34^. With the exception of osteocytes within cortical bone (which can be identified based on their location and morphology) accurate delineation of individual cells and the identification cell type is not possible following application of COX/SDH, especially when viewing closely packed cells residing within trabecular bone. Thus emerges the necessity to develop an alternative and improved method for quantifying mitochondrial respiratory chain components in bone.

Processing bone for the purpose of immunofluorescence requires consideration of several technical factors. Adequate fixation of bone tissue is imperative to prevent autolysis and maintain morphology of the tissue during the prolonged process of EDTA decalcification. 10% NBF is the most commonly used fixative in laboratory practice, although there is no recommended standard in the literature regarding required fixation times for whole bone, the penetration of which by fixatives is hindered by the hard outer cortex, relying predominantly on Volkmans canals which provide communication between the periosteum and endosteum. Different fixation time of 24, 48 and 72 hours using mouse femurs were trialled and it was observed that with fixation times less than 72 hours, a loss of signal intensity occurs with a fall in signal to noise ratio in all 4 channels when decalcification time was increased by 7 days or decalcification temperature was increased from 4 °C to room temperature. This would suggest that mouse femur is not adequately fixed after immersion in 10% NBF for 24 or 48 hours, the assumption being that loss of signal following these lower fixation times is due to degradation of target epitopes.

The method of decalcification is also of vital importance with the use of acid based decalcifying agents being detrimental, leading to increased tissue autofluorescence, increased precipitation of secondary fluorophores within the tissue compared to EDTA decalcification and inactivation of tartrate resistant alkaline phosphatase (TRAP) enzyme activity, rendering any requirement for TRAP staining of the tissue impossible. Although acid decalcification is much faster, our preference was for the use of 14% tetra-EDTA decalcification for 21 days at 4 °C which provided adequate decalcification, allowed easy microtomy and preserved morphology of target epitopes.

Antigen retrieval can be particularly problematic with formalin fixed, paraffin embedded bone due to the fragile nature of the tissue and its tendency to separate from the slide. Adequate fixation and decalcification helps to counteract this, as does allowing sections adequate time to dry following microtomy. Pressure cooker or microwave heating mediated antigen retrieval is not suitable due to the tendency of bone tissue to separate from slides under these conditions. Immersion of sections in buffer solution at temperatures greater than 80 °C also caused tissue loss; limiting the temperature to 80 °C for 30 minutes using a water bath proved effective. 1 mM tetra-EDTA was the most effective buffer for this process. It should also be noted that the form of EDTA used for antigen retrieval is of great importance with mono EDTA and di-EDTA being comparatively poor, especially for NDUFB8 epitope retrieval. Enzyme antigen retrieval had a detrimental effect on tissue morphology and was not as efficient at unmasking the mitochondrial epitopes of interest compared to heat induced epitope retrieval methods. Overnight incubation of primary antibodies at 4 °C and subsequent 2 hour incubations with secondary antibodies at the same temperature provided a more homogenous signal throughout the entirety of the tissue section and increased signal to noise ratios.

Bone is naturally a very autofluorescent substance particularly when illuminated with light emanating from the near visible ranges of the spectrum, primarily due to collagen. This leads to a reduction in the signal to noise ratio of images obtained and steps are required to counteract this. We have been unable to find any previous bone studies using four channel autofluorescence, possibly because of this difficulty. The use of an epi-fluorescent light source as opposed to a laser point scanning confocal microscope exacerbates the problem significantly, yet the use of the latter is mandatory for high quality imaging and accurate assessment. Immersing sections in 0.25% ammonia/70% ethanol was successful in reducing autofluorescence. This is a method initially introduced by Kardasewitsch in ref. 35 and verified by Baschong *et al*.^33^ more recently for use in formalin fixed paraffin embedded tissue sections being thought to reduce the autofluorescence by reacting with free formaldehyde residues and dissolving lipid derivatives^33^,^35^. Regardless of the steps taken to reduce autofluorescence, it cannot be eliminated completely, which requires lower exposure times when using a fluorescent light source or lower laser powers when using a confocal microscope to reduce background signal levels. We found the combinations of secondary antibodies imperative to generate adequate signal to noise ratios, with the relatively high substrate of porin providing excellent signal to counteract autofluorescence in the 405 emission wavelength channel and a biotinylation step being required to generate NDUFB8 signal in the 647 channel.

We used a semi-automated method of quantification in which software identifies not just cells of interest but also mitochondrial syncytium within them, and provides signal densitometry values for expression of porin, COX-I and NDUFB8 in each cell. The values for COX-I and NDUFB8 are recorded relative to signal intensity of porin (mitochondrial mass). The ability to incorporate an antibody to target porin is invaluable and provides a more accurate means of quantifying COX-I and NDUFB8 expression, removing problems of intra- and inter- observer variability seen with the method of COX/SDH histochemistry.

Application of this novel quadruple immunofluorescence protocol to formalin fixed, paraffin embedded bone tissue allowed morphology preservation, clear delineation of cells, and quantification of respiratory chain subunits within. The method has shown for the first time that osteoblasts are a cell type that is vulnerable to mutations in a mitochondrial mutator mouse model, with significant COX-I and NDUFB8 deficiencies observed at 11 months in PolgA^−/−^ mice. It is also of note that data from wild type mice aged 11 months show evidence of respiratory chain deficiency compared to their younger counterparts. Both of these findings are of interest as they provide evidence for a potentially significant role in increasing mitochondrial dysfunction occurring with age at a cellular level and potentially diminishing bone mineral density, a universal process associated with ageing.

In conclusion, our data shows that this quadruple immunofluorescence method is superior to standard COX/SDH histochemistry for use in bone as it eliminates much of the variability in result interpretation and can easily be applied to formalin fixed paraffin embedded sections of bone, allowing clear delineation and characterisation of the various cell types present. We have used the method to demonstrate respiratory chain deficiencies in the osteoblasts of aged and PolgA^−/−^ mice. Diminishing respiratory chain function seen in osteoblasts may play a significant role in the pathogenesis of bone loss with age.

## Additional Information

**How to cite this article**: Dobson, P. F. *et al*. Unique quadruple immunofluorescence assay demonstrates mitochondrial respiratory chain dysfunction in osteoblasts of aged and PolgA^−/−^ mice. *Sci. Rep.* **6**, 31907; doi: 10.1038/srep31907 (2016).

## Supplementary Material

Supplementary Information

## Figures and Tables

**Table 1:** Numerical data for individual mice used in osteoblast study, lettering corresponding to graphs in Fig. 7 and Supplementary Data S1.

	Mouse	NDUFB8 osteoblasts					COX-I osteoblasts				
		Pos	Int (+)	Int (−)	Neg	z-score	Pos	Int (+)	Int (−)	Neg	z-score
4 month wild type mice	A	100.0%	0.0%	0.0%	0.0%	−0.2645	100.0%	0.0%	0.0%	0.0%	0.1219
	B	100.0%	0.0%	0.0%	0.0%	0.4862	98.0%	2.0%	0.0%	0.0%	−0.2462
	C	100.0%	0.0%	0.0%	0.0%	−0.0199	100.0%	0.0%	0.0%	0.0%	0.0991
	D	99.2%	0.8%	0.0%	0.0%	−0.1050	100.0%	0.0%	0.0%	0.0%	−0.0740
	E	100.0%	0.0%	0.0%	0.0%	−0.1375	100.0%	0.0%	0.0%	0.0%	−0.0057
11 month wild type mice	F	61.5%	25.0%	8.7%	4.8%	−2.5793	85.6%	8.7%	2.9%	2.9%	−1.7644
	G	65.1%	25.6%	8.1%	1.2%	−2.3037	46.5%	21.0%	19.8%	12.8%	−3.1797
	H	90.1%	8.9%	1.0%	0.0%	−1.0015	87.1%	7.9%	3.0%	2.0%	−1.0838
	I	69.3%	24.8%	3.9%	2.0%	−2.3267	82.2%	13.9%	1.0%	3.0%	−1.6834
	J	69.0%	17.7%	10.6%	2.7%	−2.2288	74.3%	17.7%	7.1%	0.9%	−1.5172
	K	94.3%	5.7%	0.0%	0.0%	−0.7150	72.7%	14.8%	9.1%	3.4%	−1.4310
	L	77.9%	9.5%	4.2%	8.4%	−1.6644	67.4%	21.1%	9.5%	2.1%	−2.0048
	M	44.4%	50.0%	5.6%	0.0%	−3.1335	75.0%	19.4%	4.6%	0.9%	−1.7746
11 month PolgA mice	N	1.9%	3.9%	8.7%	85.5%	−9.0776	12.6%	8.7%	5.8%	72.8%	−10.9406
	O	3.4%	10.3%	8.0%	78.2%	−8.9053	3.4%	3.4%	2.3%	91.0%	−15.9127
	P	0.0%	0.9%	4.6%	94.4%	−9.1844	3.7%	4.6%	2.8%	88.9%	−14.9438
	Q	2.8%	0.9%	5.6%	90.7%	−9.7061	6.5%	4.6%	2.8%	86.1%	−14.1500
	R	3.0%	12.1%	24.2%	60.6%	−6.6788	12.1%	5.1%	9.1%	73.7%	−10.0046
	S	6.7%	10.6%	18.3%	64.4%	−6.6901	3.8%	5.8%	1.9%	88.5%	−13.3303
	T	0.9%	1.7%	1.0%	90.3%	−8.1441	9.6%	0.9%	3.5%	86.0%	−12.7954
	U	5.1%	14.7%	38.2%	42.0%	−5.7412	5.1%	5.1%	3.7%	86.0%	−12.1565
	V	4.1%	0.8%	4.9%	90.2%	−9.9374	4.9%	0.8%	3.3%	91.1%	−17.1963

Percentages of osteoblasts for individual mice which are positive, intermediate positive, intermediate negative and negative for NDUFB8 and COX-I protein expression are shown. Osteoblasts were classed as having normal levels (positive) if Z-scores were no more than −3SD from the mean, intermediate (+) if Z-scores were between −3SD and −4.5SD, intermediate (−) if Z-scores were between −4.5SD and −6SD, and deficient if Z-scores were more than −6SD below the mean. Average Z-scores for NDUFB8:porin and COX-I:porin are also shown.
