# Supplementary material for: Unique quadruple immunofluorescence assay demonstrates mitochondrial respiratory chain dysfunction in osteoblasts of aged and PolgA−/− mice
Source: Sci Rep. 2016 Aug 24;6:31907. doi: 10.1038/srep31907 (PMC4995399; doi:10.1038/srep31907)
Supplement: Supplementary Information [file srep31907-s1.pdf]

Unique quadruple immunofluorescence assay demonstrates mitochondrial respiratory chain dysfunction in osteoblasts of aged and PolgA$^{-/-}$ mice.

Philip F Dobson[1], Mariana C. Rocha[1], John P. Grady[1], Alexia Chrysostomou[1], Daniel Hipps[1], Sharon Watson[3], Laura C. Greaves[1,2], David J. Deehan[4,*] and Doug M. Turnbull[1,2,*]

Fixation time 10% NBF	mouse	Decalcification 14% EDTA Duration/temp	Avg. bg 405	Avg. Signal 405	405 S/N ratio	Avg. bg 488	Avg. Signal 488	488 S/N ratio	Avg. bg 647	Avg. Signal 647	647 S/N ratio
24 hours	1	18 days 4℃	247.4	1358.2	5.5	174.4	518	3.0	213.1	612.8	2.9
		18 days RT	376.4	857.6	2.3	173.6	279.7	1.6	211	269.0	1.3
	2	25 days 4℃	292.1	1626.1	5.6	219.6	933.9	4.2	275.6	1039.4	3.8
		25 days RT	301.9	1190.5	3.9	172.5	467.6	2.7	171.6	392.1	2.3
48 hours	3	18 days 4℃	232.1	1395.0	6.0	143.4	451.0	3.1	185.9	527.7	2.8
		18 days RT	386.9	1184.9	3.0	131.2	387.2	3.0	187.1	387.5	2.1
	4	25 days 4℃	312.2	1283.8	4.1	169.5	655.1	3.9	162.4	555.0	3.4
		25 days RT	448.1	1044.3	2.3	250.8	565.0	2.3	235.0	618.7	2.6
72 hours	5	18 days 4℃	199.6	1068.7	5.3	146.2	567.0	3.9	188.0	684.5	3.6
		18 days RT	236.0	1367.9	5.8	145.2	569.5	3.9	186.3	622.7	3.3
	6	25 days 4℃	256.2	1657.2	6.5	139.1	481	3.4	156.4	517.2	3.3
		25 days RT	189.4	1426.3	7.5	177.5	605.5	3.4	177.2	705.1	4.0

Supplementary Table S1. Fixation vs decalcification optimisation.

Femurs from 6 wild type mice were used to study the effects of fixation time and decalcification method, on average (avg.) background (bg) fluorescence and average positive signal pixel intensities in each of the wavelengths required for quantification (405, 488 and 647 nm). One femur from each mouse was decalcified at 4℃ and the other at room temperature (RT) in 14% tetra-EDTA pH 7.4. The solution was changed 3 times per week. 100 osteoblasts from each femur were imaged and analysed following application of the quadruple immunofluorescence assay. Following fixation at 24 or 48 hours in 10% NBF, a drop in signal to noise ratio (S/N) was observed in all 3 channels when decalcification was performed at a higher temperature, due to a consistent drop in average signal intensity and increased background fluorescence. S/N ratio was maintained following fixation at 72 hours when decalcification temperature was increased, suggesting adequate fixation of mouse femur only occurred at 72 hours.

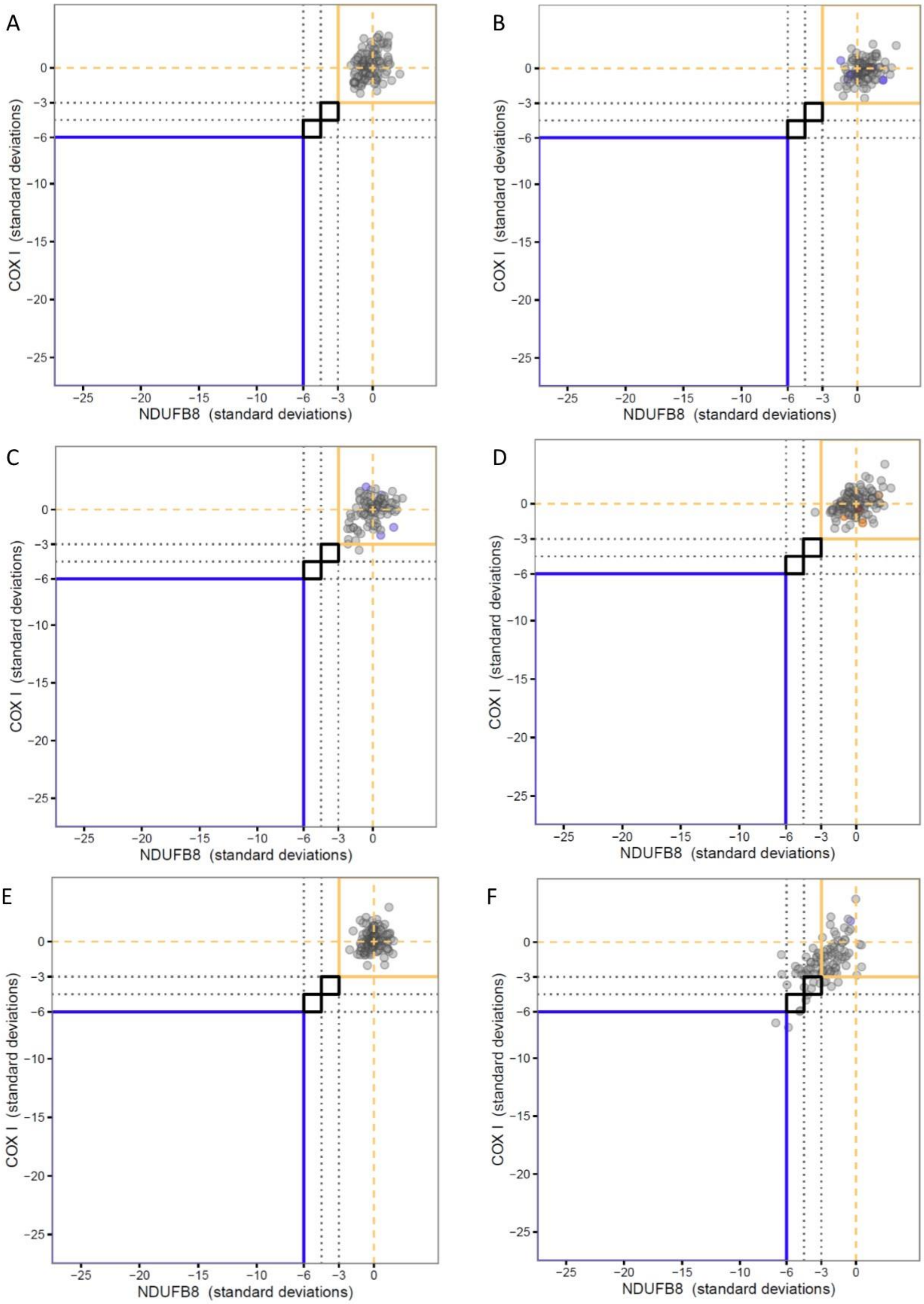
A
B
C
D
E
F
COX I (standard deviations)
NDUFB8 (standard deviations)
0
-3
-6
-10
-15
-20
-25

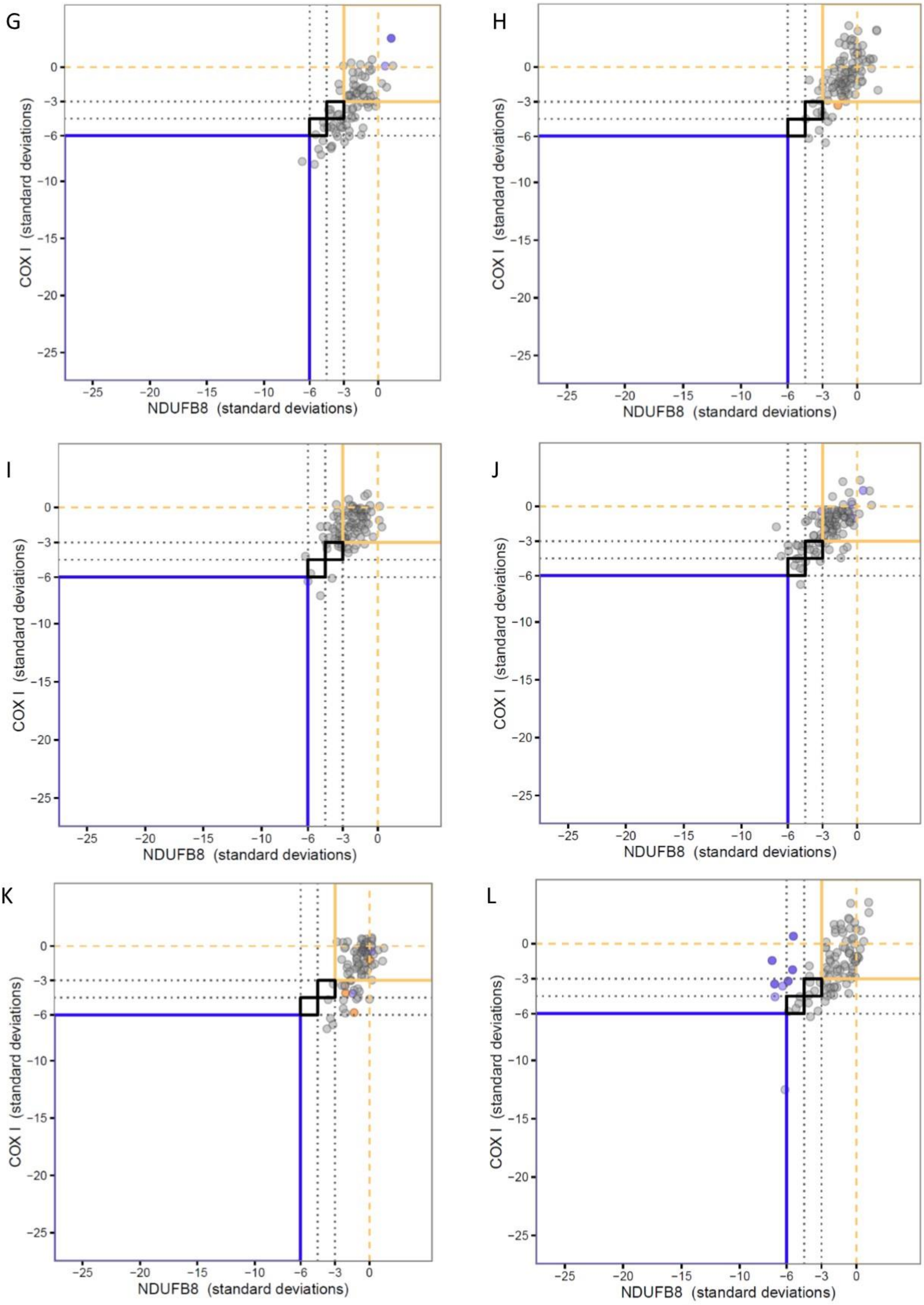
G
H
I
J
K
L
COX I (standard deviations)
NDUFB8 (standard deviations)
0
−3
−6
−10
−15
−20
−25

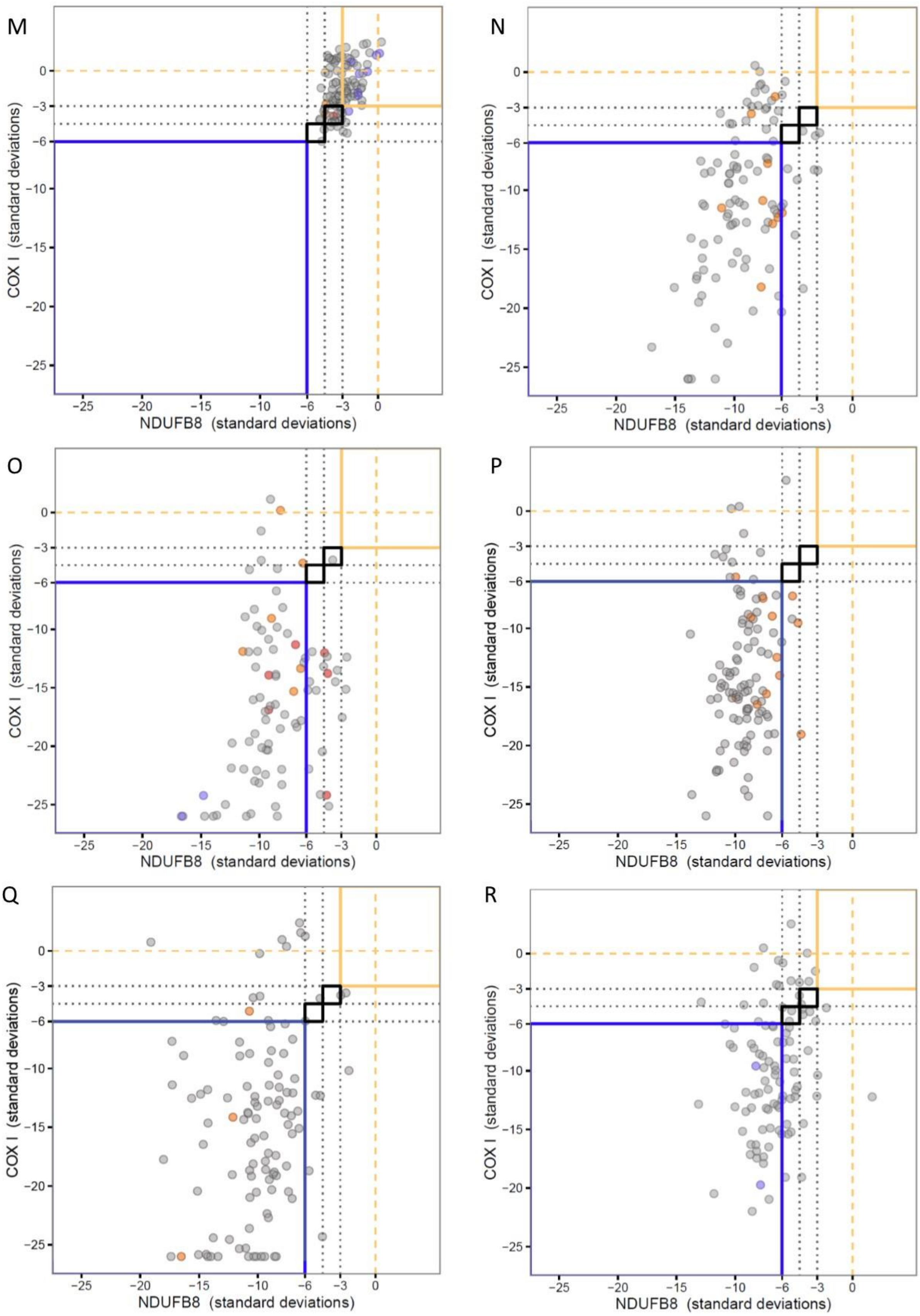
M
N
O
P
Q
R
COX I (standard deviations)
NDUFB8 (standard deviations)
0
-3
-6
-10
-15
-20
-25

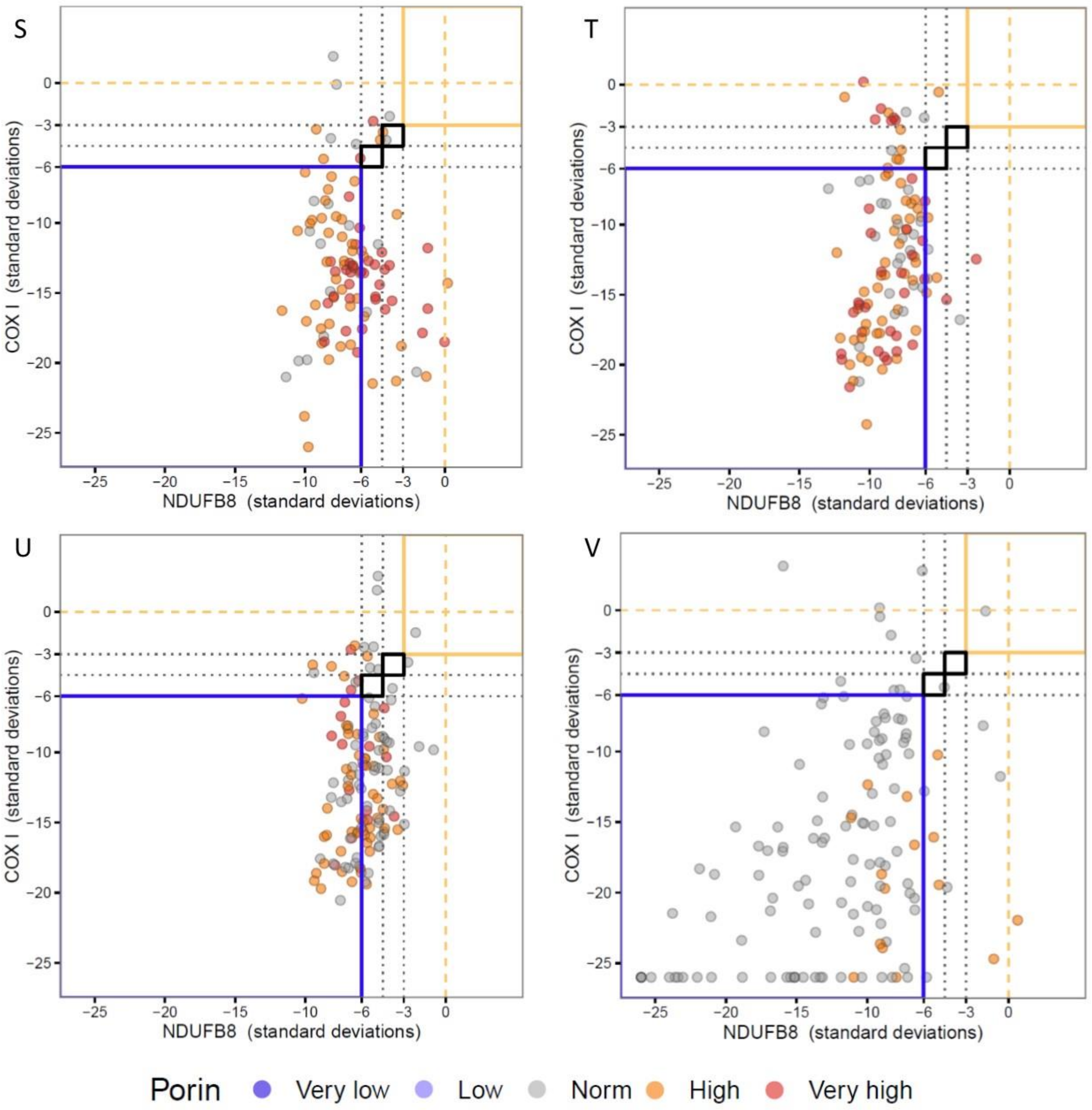

Supplementary Data S2. Individual Z-scored NDUFB8:Porin and COX-I:Porin graphs for all mice used in study.

The mean and standard deviations of NDUFB8:porin and COX-I:porin relationships in the 4 month wild type controls are established to derive Z–scores for porin, NDUFB8 and COX-I. Z-scores for NDUFB8:porin and COX-I:porin are then plotted against each other for individual mice. Each dot represents a single osteoblast, colour coded by the porin level (dark purple, very low; light purple, low; grey, normal; orange, high; red, very high). A-E represents data from 4 month old wild type animals where the vast majority of data points are no more than 3SD from the mean of this young control group. F-M shows 11 month wild type animals and N-V represents 11 month PolgA$^{-/-}$ animals with increasing deviation from the young controls, demonstrating increasing NDUFB8 and COX-I deficiencies.
